# Implicit leadership and multidimensional imitation: a structural pathway model among Chinese university students

**DOI:** 10.3389/fpsyg.2026.1794297

**Published:** 2026-05-11

**Authors:** Jinkai Zhang, Qiuling Wang, Shengmin Zhou, Lingyu Zhai, Zhuotao Fang, Gina S. Rhee

**Affiliations:** 1 School of Literature and Media, Minzu Normal University of Xingyi, Xingyi, China; 2 College of Foreign Languages, North China University of Science and Technology, Tangshan, China; 3 School of Educational Sciences, Minzu Normal University of Xingyi, Xingyi, China; 4 Catholic College, The Catholic University of Korea, Bucheon, Republic of Korea

**Keywords:** cognitive and emotional imitation, higher education, implicit leadership perception, peer learning mechanisms, social imitation, structural equation modeling

## Abstract

**Introduction:**

This study examines the association between implicit leadership perception (ILP) and social learning and imitation processes among university students in group-based educational contexts. Integrating implicit leadership theory (ILT) with the perspectives of social learning and emotional contagion, this study conceptualizes imitation as a multilevel construct that encompasses cognitive, behavioral, decision-related, and emotional dimensions through which non-formal leadership influence is manifested within peer groups.

**Methods:**

Survey data were collected from 912 Chinese undergraduate students and analyzed using a sequential analytic strategy. Confirmatory factor analysis (CFA) was primarily employed to validate the theoretically specified measurement structure, complemented by exploratory factor analysis using principal axis factoring in combination with parallel analysis. Principal component analysis was conducted only as an auxiliary technique to examine variance distribution rather than as a factor extraction method. Structural equation modeling with robust maximum likelihood estimation was then used to examine the structural relationships between ILP and four forms of imitation. Additional exploratory multigroup analyses were conducted to examine heterogeneity by role status and gender.

**Results:**

The results supported a stable five-factor structure that comprises ILP, cognitive imitation, behavioral imitation, team decision imitation, and emotional imitation. Structural analyses indicated that ILP was significantly associated with all four dimensions of imitation, with the strongest association observed for cognitive imitation, followed by team decision and emotional imitation, while a comparatively weaker association was found for behavioral imitation. The dimensions appear to be hierarchically related, although the present cross-sectional design does not allow temporal sequencing to be established. Group comparisons further highlighted systematic differences in patterns of imitation across social roles and gender.

**Discussion:**

The findings advance the current understanding of the influence of leadership within educational settings by demonstrating that imitation is not a unitary response; instead, it is a layered social learning process primarily related to cognitive mechanisms. By extending ILT beyond formal organizational contexts, the study highlights the manner in which nonexplicit leadership cues are associated with peer interaction, collective norms, and participation in classroom groups. The results provide theoretical and methodological contributions to research on social learning, leadership without authority, and group dynamics in higher education.

## 1 Introduction

In human social interaction, imitation is one of the most fundamental mechanisms of learning and adaptation. It is not only an individual-level learning strategy but also a foundational form of social influence within groups. Individuals adjust their actions after observing the behaviors and emotional reactions of others, thereby achieving social alignment and a sense of group belonging (Cialdini and Goldstein, 2004). During the formative stage of value systems and behavioral patterns, imitation is particularly prevalent among university students. When faced with academic competition, social comparison, and peer pressure, imitation becomes an important means through which students adapt to the environment (Wentzel et al., 2009). The learning strategies, expressive styles, lifestyles, and even emotional responses of peers can subtly and persistently influence one’s behavior. This influence is not typically dependent on explicit authority or commands; instead, it is realized through implicit learning and psychological identification within seemingly egalitarian peer relationships (Veenstra and Laninga-Wijnen, 2022; Mendoza and King, 2022).

The research on traditional leadership has primarily focused on the traits and behaviors of formal leaders (Bass and Avolio, 1994). However, in recent years, studies have demonstrated that influence does not originate exclusively from formal positions. In group interactions, certain individuals—despite the absence of official roles—can still influence others’ attitudes and behaviors through conduct, emotional contagion, and value demonstration (Lord et al., 2020). This phenomenon is conceptualized as *implicit leadership*. Implicit leadership theory (ILT) posits that individuals hold internalized social schemas of *leadership prototypes*. When a group member’s traits or behaviors match these schemas, other members may instinctively perceive this member as a leader and engage in imitation, compliance, or identification (Billsberry and O'Callaghan, 2024). Within university student groups, *implicit leaders* are typically characterized by academic excellence, confident self-expression, social engagement, and strong emotional influence. Even in the absence of formal authority, these individuals can guide others’ behavior through modeling effects and social referencing (Madtha et al., 2022).

Social learning theory (SLT; Bandura, 1986) conceptualizes imitation as a core mechanism through which individuals acquire behaviors, strategies, and emotional responses by observing others. In contrast, implicit leadership theory (ILT) explains how certain individuals are cognitively categorized as “leaders” based on internalized leadership prototypes (Lord et al., 1984). The integration of these two perspectives provides a mechanism-based explanation of imitation in peer contexts. Specifically, ILT addresses the question of “who becomes a model for imitation,” whereas SLT explains “how imitation occurs once a model is identified.” When a group member matches others’ implicit leadership schemas, this individual is more likely to be perceived as a salient and credible model. This perception increases observational attention and enhances cognitive alignment, which is associated with higher levels of behavioral and emotional imitation, as predicted by SLT.

Therefore, imitation among university students is not random but is selectively directed toward individuals who are implicitly recognized as leaders. In this sense, implicit leadership perception functions as a cognitive filtering mechanism that structures the direction and intensity of social learning processes within peer groups. From this integrated perspective, imitation can be understood as a structured and leader-oriented social learning process, rather than a generalized or undifferentiated response.

## 2 Literature review

### 2.1 Implicit leadership theory and leadership perception in peer contexts

ILT posits that individuals develop internal prototypes of an *ideal leader*, which can be activated when they encounter specific persons and subsequently form judgments about leadership qualities (Lord et al., 1984). Offermann et al. (1994) and Offermann and Coats (2018) proposed and empirically validated an eight-dimension prototype model, further demonstrating that these prototypes exhibit dynamic flexibility across cultural contexts.

In educational and peer learning settings, leadership roles are typically not formally designated; instead, they emerge naturally through group interaction and performance. Previous studies illustrated that individuals within peer groups may instinctively identify *natural leaders* or *central figures* and adopt them as reference points, thereby forming leader–follower relationships (Lawson and Fleshman, 2020). Schyns et al. (2012) emphasized that individuals’ awareness of their implicit leadership prototypes can be enhanced through ILT-based instruction and reflective training, which consequently facilitates more effective interaction within teams.

Recent ILT/IFT research has extended the field to shared leadership, dynamic prototypes, situational variability, and agent-based modeling (Epitropaki et al., 2023; Hesmert and Vogel, 2023). The authors also emphasized that ILT/IFT should not be viewed as a static cognitive structure but as one that can evolve through interactional processes. Furthermore, Escobar Vega et al. (2025) investigated how individuals in team and organizational contexts gradually *learn* the concept of leadership through interactive experiences and continuously reconstruct their implicit leadership prototypes in the process. Their findings indicated that ILT is not fixed at birth but is continually calibrated and formed through social interaction.

### 2.2 Social learning and imitation mechanisms (including recent research on emotional contagion and mimicry)

Bandura’s (1977) social learning theory emphasizes that individuals acquire knowledge and behavior not only through direct experience but also through observation, imitation, and vicarious reinforcement. As a core mechanism of social learning, imitation is manifested not only at the level of overt behavior but also across cognitive and emotional domains. At the cognitive level, imitation denotes the internalization and mapping of others’ thinking strategies, problem-solving pathways, and implicit rules (Wang et al., 2015). At the behavioral level, imitation is manifested in the reproduction of actions, languages, or expressive styles, thereby facilitating behavioral alignment (Byrne, 2005). At the emotional level, imitation—frequently referred to as emotional contagion—pertains to the process by which group members’ emotional states converge with those of others unconsciously or semi-consciously (Barsade, 2002; Lin et al., 2024).

Norscia and Palagi (2011) demonstrated that emotional mimicry is closely associated with empathy and constitutes a key mechanism for the maintenance of social bonds. Barsade (2002) was among the first studies within organizational behavior research to experimentally verify the *emotional contagion effect*, illustrating that team members’ emotions can spread through nonverbal cues and influence overall group performance. Barsade et al. (2018) further argued that emotional contagion follows a clear causal chain: individuals pay attention to, perceive, and imitate others’ emotional expressions, thereby achieving affective convergence and synchrony. This mechanism is also evident in educational groups—enhancing classroom cohesion and fostering a positive learning climate (Fischer et al., 2012). Therefore, from the social learning perspective, imitation should not be interpreted merely as passive behavioral reproduction but as a crucial social mechanism that links individual cognitive processing, emotional regulation, and group interaction.

A recent research in the field of emotional contagion, Marx et al. (2024) proposed a balanced self-report measure for distinguishing susceptibility to positive versus negative emotional contagion. The findings indicated that susceptibility to positive emotional contagion is associated with interpersonal functioning and prosocial tendencies, while susceptibility to negative emotional contagion is more closely linked to mental health problems. Based on the neurocognitive perspective, Wang et al. (2024) examined the influence of self-representation on emotional contagion processes and demonstrated that neural resonance mechanisms (e.g., the mirror neuron system) are more likely to trigger emotional synchrony when observers perceive greater relational closeness or similarity with others.

### 2.3 From cognition to emotion: a multilevel pathway perspective

The multilevel pathway perspective posits that the diffusion of social influence typically manifests through a progressive sequence of cognition → behavior → emotion. Dolan (2002) argued that emotion, cognition, and behavior are mutually interactive and interdependent processes. Experimental evidence from organizational behavior research further demonstrates that the emotional states of individuals within teams can spread through nonverbal signals to the broader group, thereby influencing interaction quality and collective performance.

Within classroom or peer learning contexts, the following pathway can be hypothesized: students first identify and evaluate peers’ *implicit leadership* characteristics at the cognitive level (i.e., the degree of match with their internalized schemas). They then imitate relevant strategies or expressive behaviors at the behavioral level. Finally, emotional convergence and synchrony emerge at the emotional level. Barsade et al. (2018) emphasized that individuals can achieve affective convergence—a mechanism that poses causal pathway implications at the group level—through a process of *attention–perception–imitation*.

Pinus et al. (2025) empirically demonstrated the *spillover effect* of emotion regulation: when cognitive reappraisal interventions are applied to certain group members, negative emotions among members are also significantly alleviated. This finding indicates that emotional transmission extends beyond simple contagion and may involve the diffusion of emotion regulation effects. This evidence provides contemporary empirical support for elucidating the potential amplifying or feedback functions of emotional processes through influence pathways. Similarly, Michalec et al. (2025) emphasized that mimicry and synchrony mechanisms effectively operate through various channels, including facial expression, electromyographic activity, and linguistic rhythm.

In terms of integrating implicit leadership and peer imitation pathways, the ILT processing structure model proposed by Tavares et al. (2018) is particularly informative. This model situates cognitive processing (e.g., schema activation and information filtering) at the origin of the pathway, thus elucidating the following hierarchical mechanism: implicit leadership signals → cognitive matching → behavioral imitation → emotional convergence. Moreover, recent ILT/IFT research (e.g., Epitropaki et al., 2023) conceptualized implicit prototypes as dynamically flexible structures that can be reshaped through interaction. This perspective implies that imitation pathways may not be strictly unidirectional; instead, they may involve feedback loops and processes of recalibration.

### 2.4 Research gaps and significance

Although research in higher education has increasingly paid attention to peer influence, several limitations persist. First, the majority of studies focus on overt behavioral conformity and imitation and give insufficient attention to psychological mechanisms underlying implicit leadership. Second, imitation is frequently conceptualized as a passive or negative form of social learning, thus overlooking its positive functions in fostering group cohesion, shared strategies, and emotional resonance. Third, integrative empirical frameworks for elucidating the dynamic relationships among implicit leadership, imitation mechanisms, and peer network structures are lacking.

To address these concerns, the present study integrates ILT and SLT to construct an *implicit leadership–imitation mechanism* model. This model focuses on the influence of implicit leadership on group interaction and emotional resonance through cognitive, behavioral, and emotional imitation pathways. This integrative perspective extends the theoretical limitations of the research on classroom social dynamics and provides empirical evidence relevant to the cultivation of students’ self-directed learning capacities, leadership potential, and social influence in the context of higher education.

### 2.5 Research hypotheses

Drawing on ILT and SLT and informed by a multilevel pathway perspective that spans cognition, behavior, and emotion, the present study proposes the following hypotheses:

*H1*: University students’ perceptions of implicit leadership within a group (implicit leadership perception [ILP]) will positively predict their tendency toward cognitive–academic imitation (CAI).

*H2*: University students’ ILP will positively predict their behavioral imitation tendency (BIT).

*H3*: University students’ ILP will positively predict their team conformity and decision imitation (TCDI).

*H4*: University students’ ILP will positively predict their emotional mimicry and empathy (EME).

Taken together, these hypotheses constitute the core pathway structure of the proposed *implicit leadership–imitation mechanism* model and are empirically tested in the subsequent structural equation modeling (SEM) analyses.

## 3 Questionnaire design and data collection

### 3.1 Questionnaire design

The present study used a self-developed instrument called the University Students’ Group Imitation Behavior Questionnaire (from an Implicit Leadership Perspective) as the primary measurement tool. The theoretical foundations of the questionnaire are grounded in SLT (Bandura, 1977; Bandura, 1986), social influence theory (Cialdini and Goldstein, 2004), ILT (Conger and Kanungo, 1987; Lord and Maher, 1991; Conger and Kanungo, 1994; Offermann et al., 1994), and emotional contagion theory (Hatfield et al., 1993; Barsade, 2002; Barsade et al., 2018). Collectively, these theories elucidate the manner through which individuals within social contexts achieve interaction and synchrony across cognitive, behavioral, and emotional levels through observation, imitation, and empathy, thereby providing theoretical support for the proposed multilevel pathway model.

In the present study, academically successful or socially influential peers are conceptualized as prototypical implicit leaders within university student groups. Prior research suggests that implicit leadership is often attributed to individuals who demonstrate competence, confidence, and social influence in group contexts. Therefore, items referring to “high-performing classmates” or “socially influential peers” are theoretically grounded as observable manifestations of implicit leadership prototypes. Accordingly, imitation directed toward such individuals can be interpreted as implicit leadership-oriented imitation rather than general peer imitation.

The questionnaire comprises five dimensions with a total of 45 items, which are detailed as follows:

1. ILP: based on ILT and related studies (Conger and Kanungo, 1998), this dimension measures individuals’ perceptions of *natural leaders* or informal influencers within their groups (e.g., I think that certain classmates can influence others’ behavior even without holding any formal position).
2. CAI: Drawing on SLT (Bandura, 1977) and self-determination theory (Ryan and Deci, 2000), this dimension assesses students’ tendencies toward CAI in learning strategies, goal setting, and learning motivation (e.g., I tend to imitate the time-management or learning strategies of classmates who perform well academically).
3. BIT: this dimension is adapted from the Peer Influence Scale developed by Bearden et al. (1989), supplemented by findings from social imitation research (Cook and Bird, 2011; Robinson et al., 2016). It is used to evaluate university students’ tendencies to imitate others in everyday behaviors and social activities (e.g., In social activities, I tend to imitate the behaviors of popular classmates).
4. TCDI: Informed by social influence theory and group decision-making models (Kerr and Tindale, 2004), this dimension captures imitation manifested in team discussions and collective decision-making contexts, particularly through alignment with dominant viewpoints and emerging group consensus (e.g., During group discussions, if the leader or dominant member insists on a particular viewpoint, then I usually follow it).
5. EME: Based on emotional contagion theory (Hatfield et al., 1993; Barsade, 2002) and the *chameleon effect* (Chartrand and Bargh, 1999), this dimension assesses students’ emotional resonance and emotional imitation when exposed to peers’ affective expressions (e.g., When classmates display anxiety or nervousness, I tend to experience similar emotional reactions).

All items were rated using a five-point Likert-type scale (1 = *strongly disagree*, 5 = *strongly agree*), with high scores indicating strong tendencies on corresponding dimensions. Prior to the formal survey, a small-scale pilot test was conducted to ensure item clarity and reliability. The results indicated that the Cronbach’s *α* coefficients for all subscales exceeded 0.80, which demonstrated good internal consistency.

### 3.2 Data collection

Data were collected via the online survey platform *Wenjuanxing* using a voluntary and anonymous participation procedure, thus ensuring respondents’ authenticity and data security. The questionnaire link was disseminated during the spring semester of 2025 and targeted university students nationwide. A total of 912 valid responses were obtained, yielding an effective response rate of 100%.

The sample covered students from higher education institutions across 18 provinces, including comprehensive universities, normal universities, science and engineering institutions, and ethnic minority universities, thus ensuring broad regional and institutional representativeness. The participants included students from different academic levels (freshmen to seniors, including a small number of graduate students), with a relatively balanced gender distribution. The sample also included a certain proportion of student leaders and student organization members to ensure the presence of perceivable *implicit leader* characteristics within groups. This study strictly adhered to academic ethical standards; all data were securely stored and used exclusively for academic research purposes.

## 4 Research design

### 4.1 Overall framework

This study takes *implicit leadership and imitation mechanisms among Chinese university students* as its central focus and aims to elucidate how, within informal group interactions, Chinese university students form multilevel processes of social learning and resonance—spanning cognition, behavior, and emotion—through the perception and imitation of implicit leaders. Grounded in SLT (Bandura, 1977), ILT (Lord et al., 1984), social influence theory (Cialdini and Goldstein, 2004), and emotional contagion theory (Barsade, 2002), the study proposes a multilevel pathway model that integrates cognition, behavior, and emotion. The model assumes that—within learning and social interaction contexts—university students instinctively develop perceptions of *informal influencers*, and that such implicit leadership exerts layered effects on group learning climate and emotional cohesion through the mechanisms of cognitive, behavioral, team decision, and emotional imitation.

In terms of overall design, the study follows a systematic logic from theoretical hypotheses to data modeling. It adopted a questionnaire-based quantitative approach and used SEM to empirically test the proposed multilevel pathways. The basic logic of the research framework can be summarized as follows: *theoretical hypothesis formulation → scale construction and validation → data quality assessment → multilevel pathway modeling → group difference analysis → network-based validation*. This design emphasizes methodological coherence, thus ensuring internal consistency across analytical stages.

Apart from testing the overall *implicit leadership–imitation mechanism* model, the study conducts two exploratory multigroup analyses to examine the model’s stability across subgroups. First, the participants were grouped according to whether they hold positions in class or student organizations (*Position*) to examine whether behavioral imitation pathways vary as a function of social status and visibility. Second, the participants were grouped by gender to verify whether CAI pathways differ in terms of information-processing tendencies. Given that these analyses are *post hoc* subgroup tests, their primary purpose is to determine model heterogeneity and generate hypotheses for future research, instead of making causal claims based on cross-sectional data.

**Figure 1:**
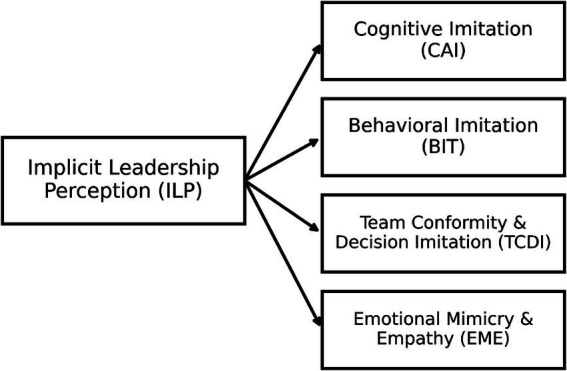
Conceptual model of the implicit leadership–imitation mechanism model.

Based on this theoretical integration, the study constructs a multilevel pathway model of the *implicit leadership–imitation mechanism* (Figure 1). In this model, ILP serves as the upstream latent variable and is hypothesized to influence social learning and emotional resonance processes among university student groups through four distinct pathways, namely, cognitive, behavioral, team decision, and emotional imitation. The conceptual model is used to elucidate theoretical assumptions and pathway structure, which are subsequently subjected to empirical testing via SEM.

### 4.2 Data analysis procedure

R was used as the primary statistical tool, and the researcher independently developed and debugged all analytical procedures to ensure the controllability and reproducibility of model estimation. The entire analytical process systematically progresses from data preparation and measurement validation to structural analysis, with each step logically structured and methodologically interdependent.

During the scale evaluation stage, Cronbach’s *α* coefficients were first computed to assess the internal consistency of each subscale. In addition, the Kaiser–Meyer–Olkin (KMO) test and Bartlett’s test of sphericity were employed to determine whether the data were suitable for factor analysis. Prior to SEM construction, the study systematically examined multicollinearity among the predictor variables to ensure the robustness of model estimation and interpretability of coefficients. Variance inflation factor (VIF) and tolerance indices were also calculated to diagnose potential collinearity issues among the key predictors.

After establishing satisfactory reliability, the study primarily employed confirmatory factor analysis (CFA) to test the theoretically specified measurement structure of the implicit leadership–imitation framework. CFA was conducted using maximum likelihood estimation with robust corrections, allowing for the evaluation of model fit and the validation of the hypothesized latent constructs (Lambert and Newman, 2023). To complement the theory-driven CFA approach and to examine the empirical dimensional structure of the data, exploratory factor analysis (EFA) was additionally performed using principal axis factoring (PAF) with oblique rotation (Oblimin). In line with the analytical strategy, parallel analysis was applied to determine the appropriate number of factors, thereby reducing the risk of over- or under-extraction and ensuring consistency between empirical patterns and theoretical expectations.

To provide an auxiliary perspective on data structure and variable clustering, principal component analysis (PCA) was conducted on standardized data. By calculating the variance contribution and cumulative explained variance of each principal component—supplemented by visualizations such as scree plots and biplots—the analysis offers an intuitive depiction of indicator clustering trends. Consistent with the analytical framework, PCA was used solely for exploratory and descriptive purposes to examine variance distribution and was not employed as a factor extraction method in either the EFA or CFA procedures.

At the structural analysis stage, the study examined the relationships among variables using composite-based structural equation modeling implemented in the lavaan package. Given the complexity of the measurement model and the large number of indicators, composite scores were computed for each construct to improve model stability and estimation reliability. The structural model specified implicit leadership perception (ILP) as the predictor variable associated with four types of imitation behaviors (cognitive, behavioral, team decision, and emotional imitation). Maximum likelihood robust estimation (MLR) was employed for parameter estimation. The results focused on standardized path coefficients and their statistical significance. All paths from ILP to the four imitation dimensions were significant (*p* < 0.001), indicating robust and consistent associations.

To facilitate intuitive interpretation, the semPlot package was used to visualize the structural relationships. Node layouts and path labels were adjusted to enhance model readability.

To further examine the applicability and stability of the model across groups, comparative analyses were conducted. Using gender (Sex) and whether participants held positions in class or student organizations (Position) as grouping variables, the study computed group differences in composite scores of the four imitation dimensions and used ggplot2 to visualize grouped bar charts and mean distribution plots. The results revealed systematic differences across groups, thus providing empirical support for subgroup-specific interpretations in Section 6.

## 5 Results

### 5.1 Reliability analysis

The study calculated Cronbach’s *α* coefficients to examine the internal consistency of each subscale. The results demonstrate that the α coefficients for the ILP, CAI, BIT, TCDI, and EME scales were 0.83, 0.92, 0.92, 0.87, and 0.81, respectively. All α coefficients exceeded the well accepted psychometric threshold of 0.70 (Nunnally and Bernstein, 1994), which indicates good internal consistency and measurement stability. In summary, the questionnaire demonstrated high reliability across different dimensions of imitation and can reliably capture the multidimensional imitation responses of university students regarding ILP.

### 5.2 Validity testing

To further verify the suitability of the scale structure and convergent validity of the latent variables, the study employed KMO measures of sampling adequacy and Bartlett’s test of sphericity for all items. The results indicated an overall KMO value of 0.966, with Bartlett’s test reaching statistical significance (*p* < 0.001), indicating that the data were highly suitable for factor analysis. The KMO values for all subscales exceeded 0.80, which further supported the rationality of the internal scale structure. These findings underscored a high degree of commonality among the five subscales and sufficient inter-item correlation to support latent factor extraction. Simply put, shared latent constructs can explain the measurements of implicit leadership and cognitive, behavioral, team decision, and emotional imitation, providing empirical support for the theoretically proposed multilevel *cognition–behavior–emotion* pathway model.

### 5.3 Multicollinearity diagnostics

Prior to SEM, linear dependency tests were conducted on all subscales and the entire dataset to avoid potential interference from multicollinearity in path estimation. The results indicated that the average VIF across all items was 2.47, with a maximum value of 3.61, and the maximum condition index (CI) was 9.28. All values were well below commonly cited warning thresholds (VIF > 10 and CI > 30). The average VIF values for the subscales ranged from 1.64 to 2.32, with no maximum VIF more than 3.36 and all maximum condition indices less than 6.

### 5.4 Exploratory factor analysis

To examine the latent structure of the questionnaire and its consistency with the theoretical dimensions, an exploratory factor analysis (EFA) was conducted using principal axis factoring (PAF) with oblimin rotation, following recent methodological recommendations. Prior to factor extraction, the Kaiser–Meyer–Olkin (KMO) value was 0.966, and Bartlett’s test of sphericity was significant (*p* < 0.001), confirming the suitability of the data for factor analysis.

#### 5.4.1 Results of parallel analysis

**Figure 2:**
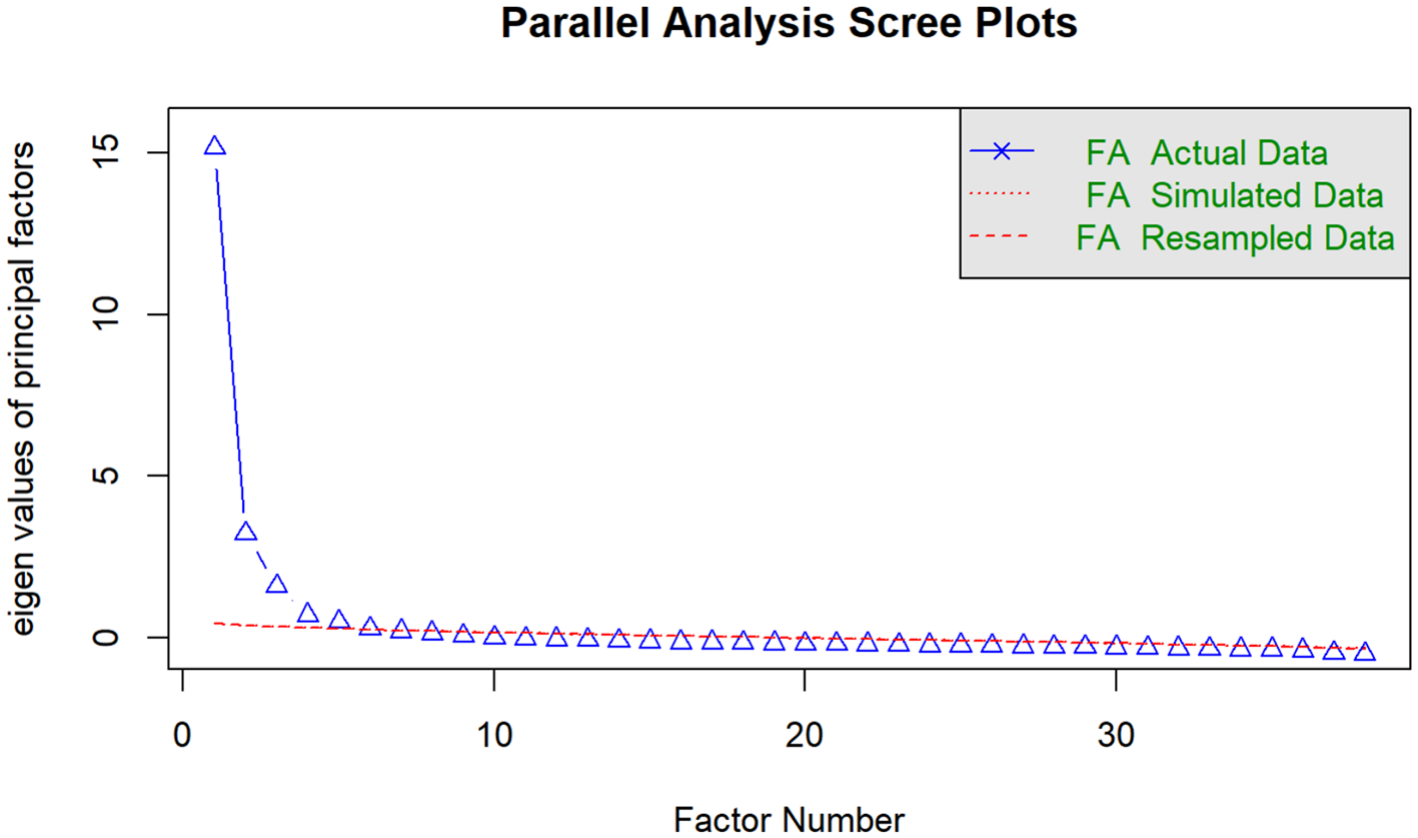
Parallel analysis for determining the number of factors.

Parallel analysis was conducted to determine the appropriate number of factors. As shown in Figure 2, the eigenvalues of the actual data exceeded those of the simulated data for the first six factors, suggesting a six-factor solution. However, the scree plot revealed a clear inflection point after the fifth factor, indicating diminishing returns in explained variance beyond this point. Although parallel analysis suggested a six-factor solution, the sixth factor showed limited explanatory value, substantial cross-loadings, and weak theoretical coherence. Therefore, a five-factor solution was retained on the grounds of interpretability and parsimony. In the present study, the sixth factor contributed relatively little variance and exhibited substantial cross-loadings, and lacked clear theoretical interpretability. Therefore, the factor structure was interpreted in light of the theoretical framework, and a more parsimonious five-factor solution was retained for interpretation. This decision is consistent with common practices in scale development, where theoretical interpretability and parsimony are considered alongside statistical criteria (Table 1).

**Table 1:** Variance explained through five-factor EFA.

Measure	ML3	ML2	ML4	ML5	ML1
SS loadings	6.0357	5.7876	3.5331	3.4344	3.3549
Proportion variance	0.1588	0.1523	0.0930	0.0904	0.0883
Cumulative variance	0.1588	0.3111	0.4041	0.4945	0.5828
Proportion explained	0.2725	0.2613	0.1595	0.1551	0.1515
Cumulative proportion	0.2725	0.5339	0.6934	0.8485	1.0000

#### 5.4.2 Factor loadings and structural patterns

**Figure 3:**
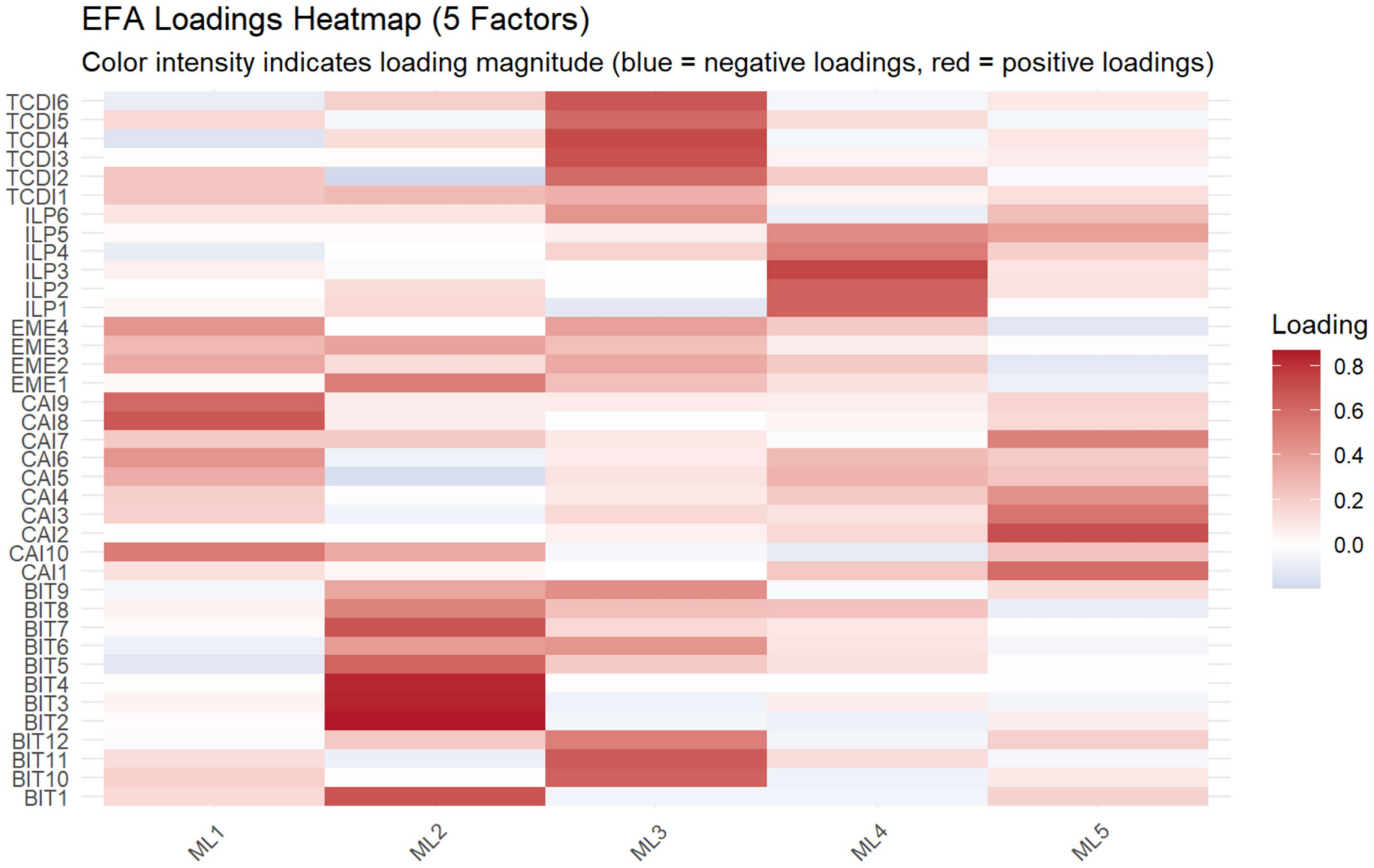
Heatmap of factor loadings.

Figure 3 presents the factor loading matrix obtained using PAF with oblimin rotation. The heatmap illustrates a generally interpretable structure, with clear clustering patterns across most items.

Based on a loading threshold of 0.40, three items (ILP6, CAI5, and TCDI1) were identified as having low factor loadings and were removed from subsequent analyses. The EFA was then re-estimated using the refined item set.

The resulting factor structure showed that ILP items primarily loaded on a single factor (PA4), with loadings ranging from approximately 0.49 to 0.74, representing perceptions of implicit leadership within the group. CAI items were mainly distributed across two closely related factors (PA3 and PA1), suggesting that cognitive imitation may involve multiple subcomponents. BIT items largely loaded on PA2, with relatively high loadings (up to 0.84), indicating a strong behavioral imitation tendency. TCDI items clustered on PA3, reflecting conformity in group decision-making contexts. EME items were primarily associated with PA5, indicating emotional contagion and affective alignment.

Although some cross-loadings were observed (e.g., BIT6, EME3, and ILP5), these patterns are expected given the conceptual relatedness among different forms of imitation. Overall, the factor structure was broadly consistent with the theoretical framework.

#### 5.4.3 Variance explained and interpretation

The extracted factors jointly explained 60.57% of the total variance. The first two factors accounted for relatively larger proportions of variance, indicating that behavioral imitation and related processes play a central role in the overall structure. The remaining factors contributed moderate but meaningful proportions of variance, supporting the multidimensional nature of imitation. Taken together, the EFA results provide preliminary support for the construct validity of the questionnaire. Given the theory-driven nature of the study, confirmatory factor analysis (CFA) was subsequently conducted to further validate the measurement model.

These findings further justify the use of CFA as a theory-driven approach to confirm the measurement structure.

### 5.5 Principal component analysis

As an auxiliary technique complementing the EFA—used here solely to examine variance distribution rather than as a factor extraction method—principal component analysis (PCA) was conducted on the 38 standardized items to further examine the aggregation characteristics and structural concentration of the questionnaire items across latent dimensions. The final sample included 912 valid questionnaires.

#### 5.5.1 Eigenvalues and variance explained

PCA was also conducted on all items to further examine the concentration and aggregation of variance across latent dimensions. Based on the Kaiser criterion (eigenvalues > 1), the study retained five principal components. Table 2 reports the eigenvalues and variance explained by each component.

**Table 2:** Standardized path coefficients and explained variance of the structural model.

Outcome	*β*	*p*	*R* ^2^
CAI	0.738	<0.001	0.456
BIT	0.533	<0.001	0.716
TCDI	0.628	<0.001	0.605
EME	0.602	<0.001	0.638

The first principal component (PC1) exhibited a dominant eigenvalue of 15.742, which accounted for 41.4% of the total variance. The second component (PC2) obtained an eigenvalue of 3.865, explaining 10.2% of variance, followed by PC3 (eigenvalue = 2.200, 5.8%), PC4 (eigenvalue = 1.343, 3.5%), and PC5 (eigenvalue = 1.069, 2.8%).

The first four components jointly explained 60.9% of the total variance, and the cumulative variance explained by the five retained components reached 63.7%—meeting the commonly accepted criteria for dimensional reduction in social science research. In summary, the PCA results indicated a highly concentrated variance structure and are primarily consistent with the five-factor solution identified via EFA, which provides additional support for the structural stability of the measurement instrument.

#### 5.5.2 Component loadings and variable aggregation

**Figure 4:**
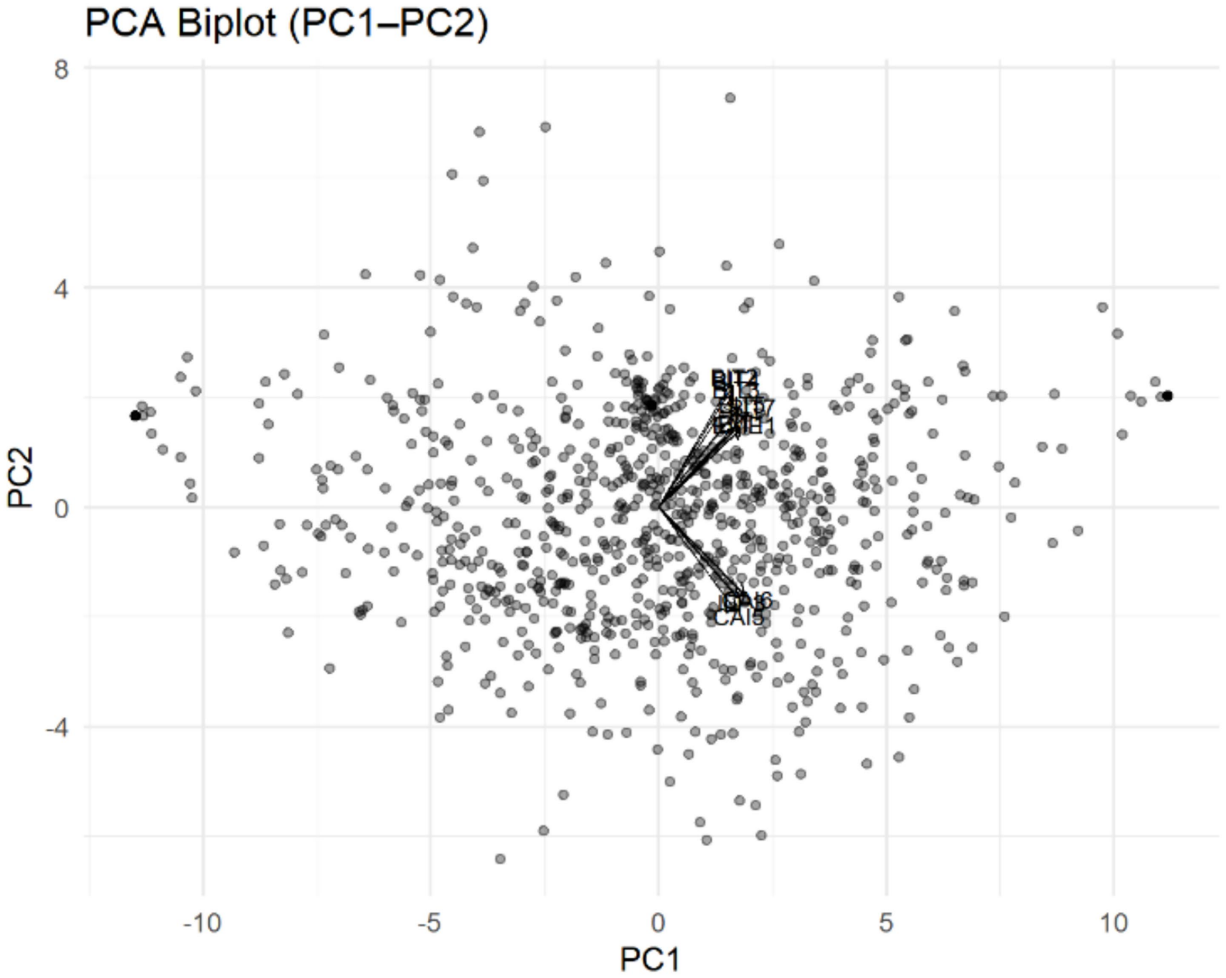
PCA biplot of the first two principal components (PC1 and PC2).

Inspection of the principal component loading matrix reveals that the first two components (i.e., PC1 and PC2) are the most representative in terms of information extraction. As illustrated in the PCA biplot (Figure 4), the distribution of observations in the PC1–PC2 plane is balanced, exhibiting clear loading directions and a high degree of concentration among variables; no significant outliers were detected.

Items on behavioral imitation (BIT2, BIT4, BIT3, BIT7, and BIT5) and cognitive imitation (CAI5 and CAI6) displayed the highest combined loadings on the first two principal components (|*r*| ≈ 0.27–0.33), which indicates that these variables contribute most significantly to the principal component structure. Meanwhile, items on implicit leadership (ILP3) and emotional imitation (EME1) reached moderate loadings on PC1 and PC2 (|*r*| ≈ 0.25)—implying that leadership perception and emotional resonance play secondary but supportive roles within group imitation mechanisms. Overall, PC1 primarily reflects BIT within social interaction, while PC2 represents cognitive and emotional imitation tendencies. Taken together, these components capture the core mechanism through which students observe, interpret, and imitate others’ behaviors within group contexts.

#### 5.5.3 Model interpretation and summary of results

Collectively, the EFA and PCA results reveal highly consistent structural patterns. The results of both analytical approaches indicate that the scale consists of five interrelated but distinct dimensions, jointly supporting the measurement framework of the proposed *implicit leadership–imitation mechanism* model. The first five principal components extracted via PCA explain more than 60% of the total variance while preserving dimensional differentiation, thus demonstrating strong information condensation and dimensional stability. These findings further support the empirical validity of the proposed three-layer imitation pathway model that spans cognition, behavior, and emotion and provide robust statistical justification for latent structure modeling in subsequent SEM analyses (Table 3).

**Table 3:** Standardized path coefficients from implicit leadership perception to the dimensions of imitation.

Path	Standardized coefficient (β)	Standard error	*z*	Significance
ILP → BIT	0.529	0.058	10.08	***
ILP → CAI	0.833	0.067	14.75	***
ILP → TCDI	0.695	0.068	11.40	***
ILP → EME	0.693	0.060	11.64	***

*** *p* < 0.001.

### 5.6 Structural model analysis

To examine the relationships between implicit leadership perception and different types of imitation behaviors, a structural model was constructed using composite variables. Composite scores were computed by averaging item responses for each construct, and these measures were subsequently used to estimate the structural relationships among variables.

The structural relationships were estimated using the lavaan package with robust maximum likelihood estimation (MLR), which provides reliable parameter estimates under non-normal conditions. In the model, ILP was specified as the predictor variable, while the four types of imitation behaviors were treated as outcome variables.

This modeling approach reduces parameter estimation complexity and enhances model stability, which is particularly suitable when constructs are measured by a relatively large number of items. Moreover, it allows for a clear examination of structural relationships among constructs without imposing additional constraints associated with complex measurement models.

#### 5.6.1 Structural model results

**Figure 5:**
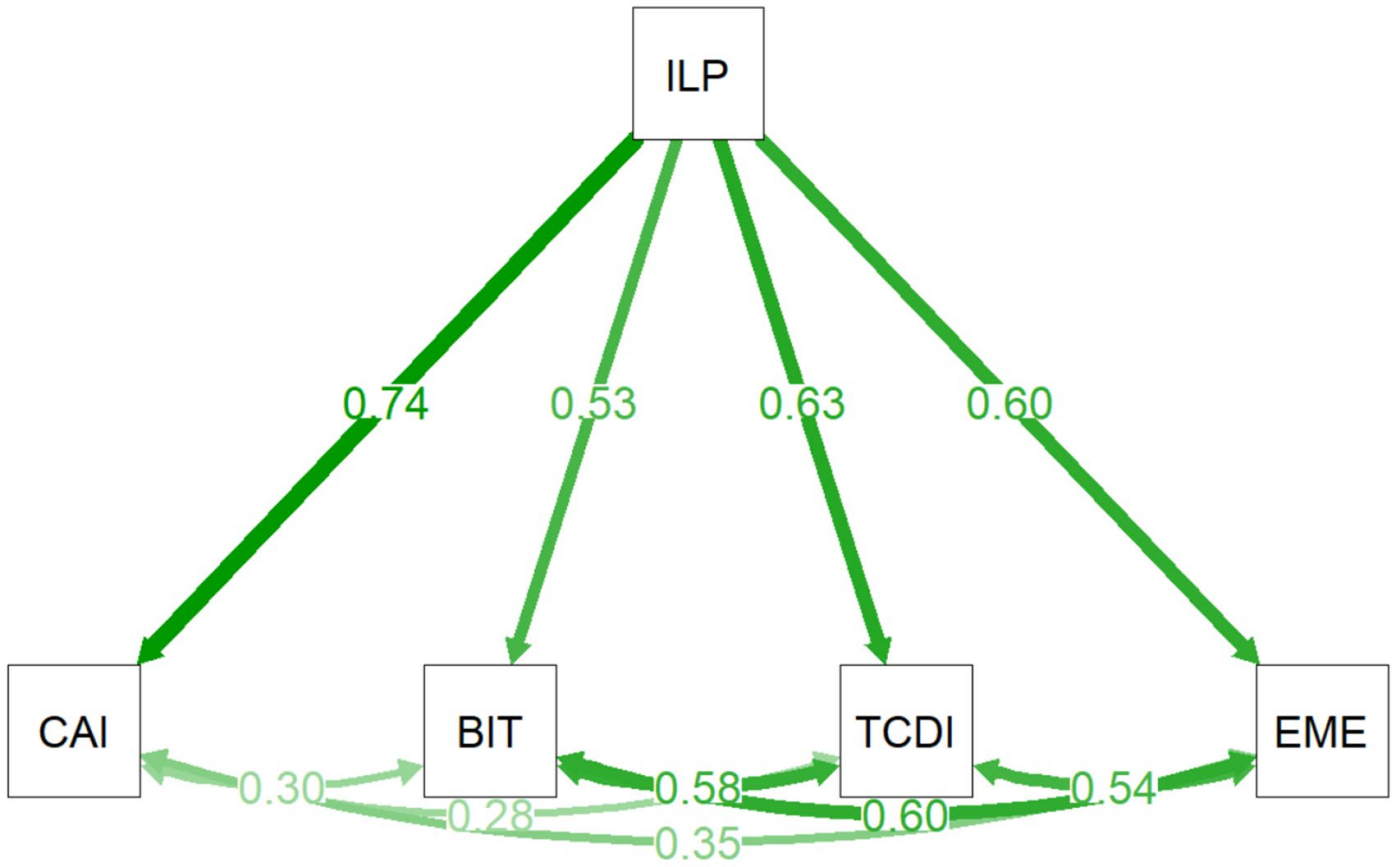
Structural relationships between implicit leadership perception and four types of imitation behaviors.

In addition, Wald tests were conducted to formally compare the magnitude of path coefficients across different imitation dimensions. As shown in Table 2 and Figure 5, implicit leadership perception (ILP) demonstrated significant positive effects on all four types of imitation behaviors (all *p* < 0.001).

Specifically, ILP had the strongest effect on cognitive imitation (*β* = 0.738), followed by team decision imitation (*β* = 0.628) and emotional imitation (*β* = 0.602), while the effect on behavioral imitation (*β* = 0.533) was comparatively weaker.

In terms of explanatory power, ILP accounted for 45.6% of the variance in cognitive imitation, 71.6% in behavioral imitation, 60.5% in team decision imitation, and 63.8% in emotional imitation.

Furthermore, moderate residual correlations were observed among the four imitation dimensions (*r* = 0.28–0.60), suggesting that while these constructs are related, they remain empirically distinguishable.

#### 5.6.2 Construct validation

Given the complexity of the measurement model and the large number of indicators, composite scores were computed for each construct (ILP, CAI, BIT, TCDI, and EME) by averaging their respective items. This approach is commonly adopted to improve model stability and reduce estimation bias in structural modeling.

Prior reliability analyses indicated that all scales demonstrated good internal consistency (Cronbach’s *α* > 0.80), supporting the reliability of the composite measures. In addition, the results of the exploratory factor analysis (EFA) provided evidence for the construct validity of the questionnaire, indicating that the measurement structure was broadly consistent with the theoretical framework.

### 5.7 Group difference analysis

While PCA results provide an auxiliary perspective on variable aggregation, the group comparisons in this section are based on composite scores of the four imitation dimensions.

**Figure 6:**
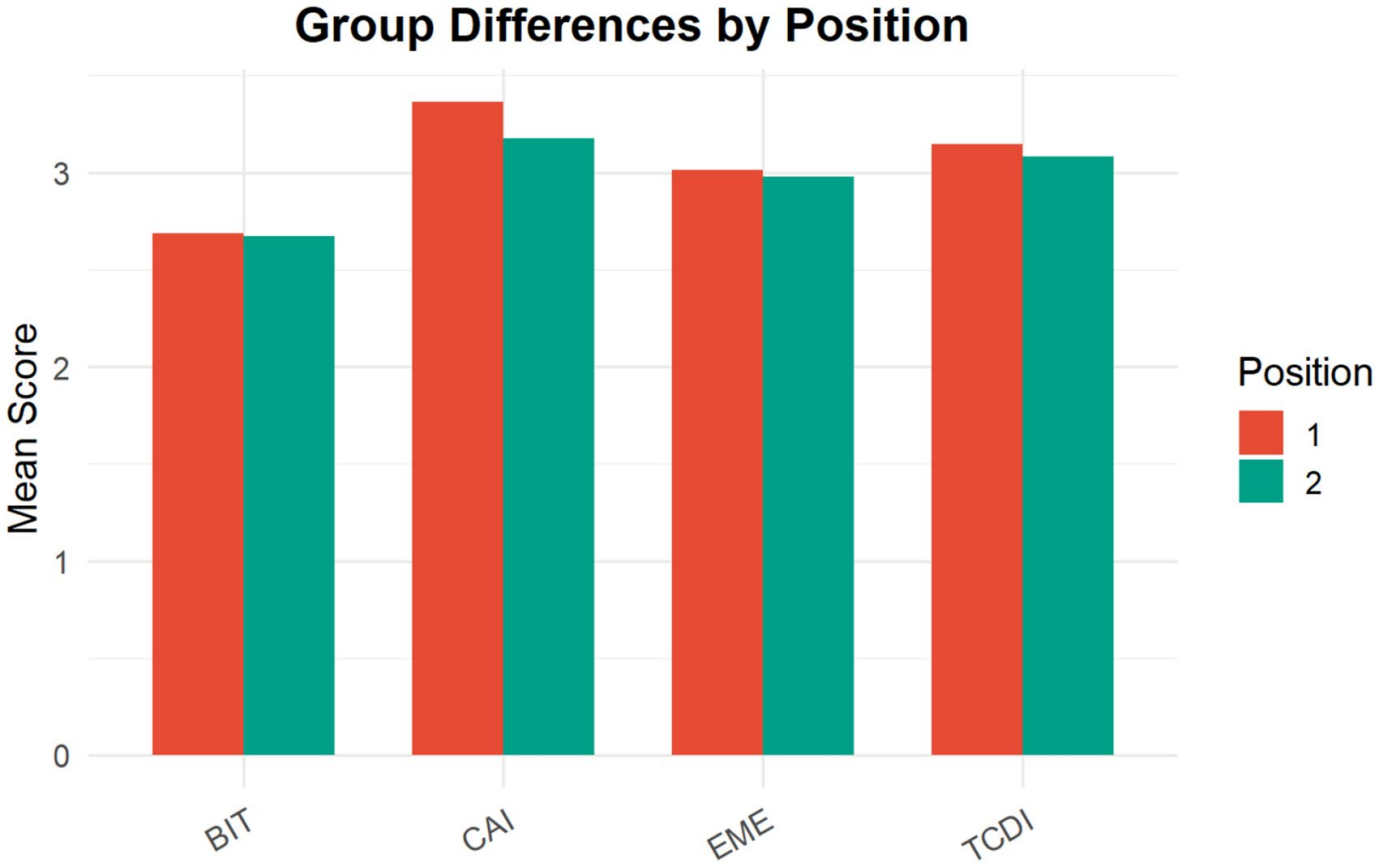
Group differences in imitation behaviors by position status.

**Figure 7:**
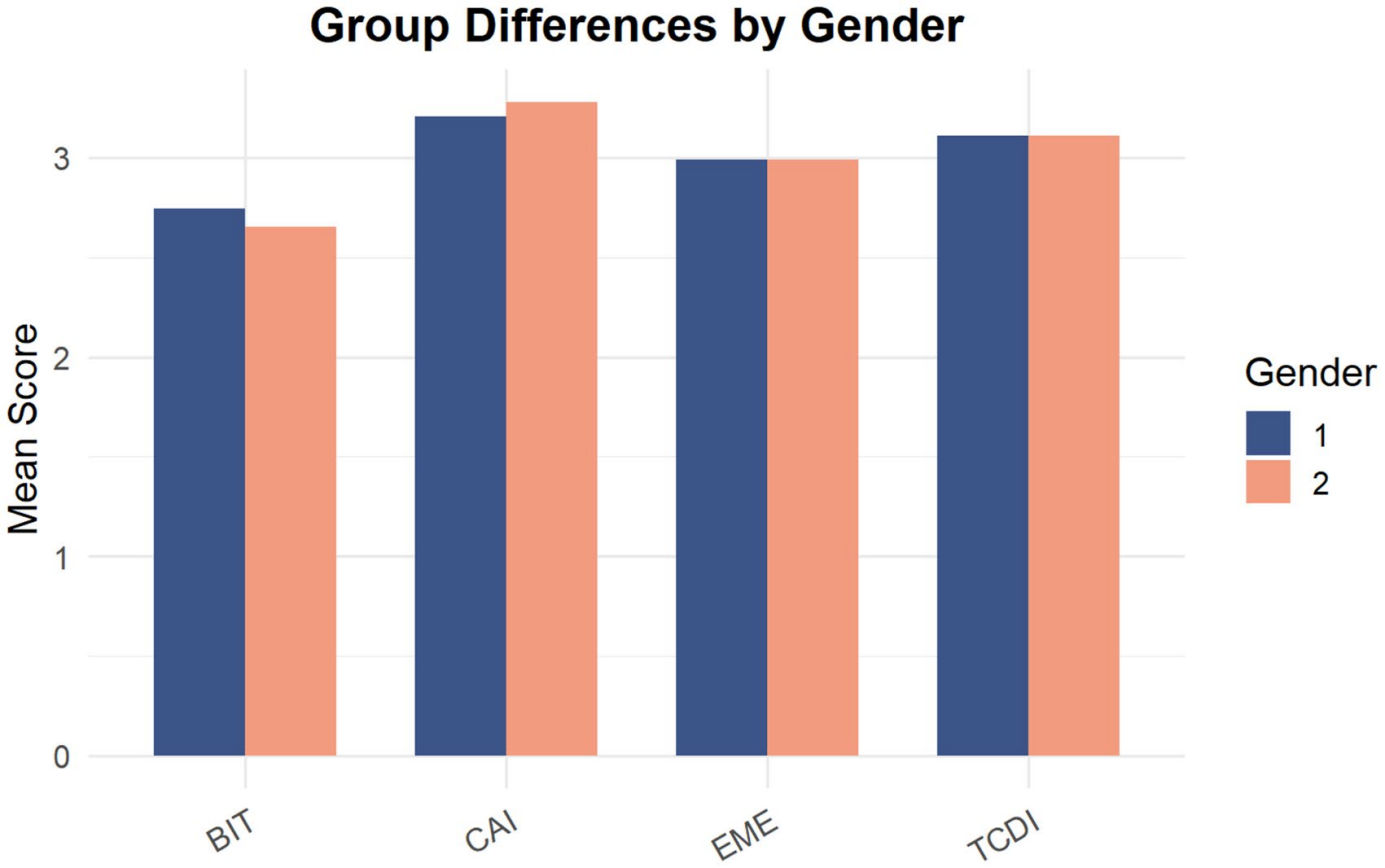
Group differences in imitation behaviors by gender.

To further examine differences across groups in terms of imitation behaviors, comparative analyses were conducted using gender (Sex) and whether participants held positions in class or student organizations (Position) as the grouping variables. Composite scores for behavioral imitation (BIT), cognitive imitation (CAI), team decision imitation (TCDI), and emotional imitation (EME) were compared across groups. Figures 6, 7 present the results, and statistical significance was evaluated using independent-samples *t*-tests.

#### 5.7.1 Differences by position (position groups)

Figure 6 presents the group differences across the four dimensions of imitation.

* Bars represent mean scores, and statistical significance was evaluated using independent-samples *t*-tests.

For cognitive imitation (CAI), the Position 1 group (holding positions in class or student organizations) exhibited a significantly higher mean score than the Position 2 group (*t* = 3.30, *p* = 0.001; *M*₁ = 3.36; *M*₂ = 3.18), suggesting that individuals occupying central or leadership roles tend to exhibit higher levels of cognitive alignment and information processing.

In contrast, no statistically significant differences were observed for behavioral imitation (BIT; *t* = 0.30, *p* = 0.77), team decision imitation (TCDI; *t* = 1.12, *p* = 0.26), or emotional imitation (EME; *t* = 0.60, *p* = 0.55), although the Position 1 group showed slightly higher mean values across these dimensions.

The position groups exhibited a modest pattern of differentiation primarily at the cognitive level, while differences in behavioral, decision-related, and emotional imitation remained limited.

#### 5.7.2 Gender differences (gender groups)

Figure 7 illustrates gender differences across the four dimensions of imitation.

The results indicated that no statistically significant gender differences were observed for behavioral imitation (BIT; *t* = 1.58, *p* = 0.12), cognitive imitation (CAI; *t* = −1.08, *p* = 0.28), team decision imitation (TCDI; *t* = −0.05, *p* = 0.96), or emotional imitation (EME; *t* = −0.01, *p* = 0.99).

Although minor variations in mean scores were observed (e.g., slightly higher behavioral imitation among males and slightly higher cognitive imitation among females), these differences were not statistically significant.

## 6 Discussion

Given that the present study is based on cross-sectional data, the findings should be interpreted as reflecting predictive associations rather than establishing causal relationships.

### 6.1 From implicit leadership perception to a multilevel imitation system: a cognitively driven pathway of influence

The present study finds that ILP is significantly associated with the four types of imitation. Among them, the strongest association is observed for CAI, followed by TCDI and EME, while BIT presents a comparatively weaker association. The pattern observed in this study—linking implicit leadership perception with cognitive following, decision/behavioral alignment, and emotional resonance—is consistent with process models in leadership theory that emphasize interactions among cognitive, behavioral, and emotional mechanisms (Walter and Scheibe, 2013). Moreover, research on emotional mechanisms and emotional contagion supports the important role of emotion in leadership-related interactions (Sy and van Knippenberg, 2021).

Students’ intuitive judgments regarding who appears to be a leader are associated with cognitive-level alignment, including strategic following behaviors (e.g., learning strategies, time management, and goal setting). These cognitive tendencies tend to co-occur with patterns of decision consensus and shared group climate in team interactions, while emotional resonance is also related to this alignment (Barsade and Knight, 2015; van Kleef and Côté, 2022). Using large-sample quantitative evidence from group contexts, the findings suggest a structured but not necessarily linear pattern of association among the four dimensions.

### 6.2 Why does behavioral imitation not take the lead? A reinterpretation from social context and cost–benefit perspectives

In contrast to the strong behavioral mimicry typically observed in dyadic interactions associated with the chameleon effect (Chartrand and Lakin, 2013), the present study shows that BIT is relatively weaker in classroom settings with numerous members. This pattern is consistent with discussions in community and group research regarding the trade-off between imitation costs and visibility. In evaluative or competitive environments, individuals may prefer low-cost and less externally visible forms of alignment—such as cognitive alignment or shared strategies—and reserve high-visibility behavioral imitation for contexts with clear norms or low interpersonal risk (Farmer et al., 2018).

Thus, in semipublic, multiobserver environments such as classrooms or student organizations, the cost structure of imitation may shift, with cognitive imitation appearing to be a safer and more preferred form of alignment.

### 6.3 Bridging role of team decision imitation: from individual processing to group norms

The path coefficient for TCDI ranks second, suggesting that consistency in team contexts does not merely reflect blind conformity. Instead, implicit leadership cues may be interpreted through shared frameworks within the group. This interpretation indicates that the responses of team members to implicit leadership cues are associated with collective meaning construction rather than mere imitation, which aligns with sensemaking theory proposed by Maitlis and Christianson (2014).

When group members reach an implicit consensus regarding who is more credible or who fits the leadership prototype, informational weighting and speaking turns in discussions may shift toward these quasi-leaders, which is associated with patterns of normative following. The significant association between ILP and TCDI is consistent with a process in which leadership prototypes are linked to both individual cognition and patterns of information flow within the team.

### 6.4 Emotional imitation is not an auxiliary variable: the consolidating role of affective climate

The findings show that ILP is significantly associated with EME, which is consistent with theoretical models that link leadership, emotional contagion, and group processes. Walter and Bruch (2008) proposed that team emotions may generate affective convergence through a positive affect spiral, while van Knippenberg and van Kleef (2016) emphasized that the emotional expressions of leaders are related to team climate and performance via emotional contagion.

In contrast to single-point measurements, this study situates EME within the broader multilevel structure, suggesting a potential dual role. On the one hand, emotional synchrony may enhance affective commitment to existing norms and goals; on the other hand, it may reduce coordination costs across interpersonal boundaries, thereby supporting the sustainability of cognitive and behavioral alignment over time. Therefore, emotion is not merely a passive outcome of leadership-related processes but also an important social component associated with the stabilization and reinforcement of alignment within groups.

### 6.5 Group roles and gender differences: who leads and who follows?

The results of multigroup comparison suggest limited and dimension-specific differences across role status and gender in the four dimensions of imitation. Individuals occupying core or leadership positions exhibit significantly higher levels of cognitive imitation (CAI), while no statistically significant differences are observed for behavioral imitation (BIT), team decision imitation (TCDI), or emotional imitation (EME).

This pattern can be interpreted from the perspective of network role differentiation and status alignment and is consistent with sensemaking theory (Maitlis and Christianson, 2014). From this perspective, higher-status individuals may demonstrate stronger tendencies toward cognitive alignment and strategic information processing, while differences in overt behavioral and emotional imitation remain relatively limited. For gender differences, no statistically significant differences are observed across the four imitation dimensions. Although minor variations in mean scores are present, these differences are not statistically meaningful and should be interpreted with caution. In this context, prior findings suggesting gender-related differences in academic performance (Voyer and Voyer, 2014) may still provide a useful background for interpreting potential variation in cognitive strategies, but such differences are not empirically supported in the present data.

### 6.6 Theoretical contributions

The theoretical contributions of this study are threefold. First, it extends ILT from organizational and workplace contexts to peer interaction among university students and proposes that implicit leadership cues represent activated social cognition rather than static traits. This perspective highlights how ILP is associated with informal learning processes in peer groups through schematic activation.

Second, this study proposes a hierarchical pattern involving cognition, behavior, and emotion, and provides empirical support for the idea that imitation is a multilayered social learning process operating across different levels, rather than a unidimensional response.

Third, this study develops a measurement system for the implicit leadership–imitation model tailored to university contexts and establishes preliminary evidence of instrument validity through EFA, PCA (as an auxiliary analysis), and SEM. This approach provides a reusable measurement framework for future cross-cultural and interdisciplinary research.

## 7 Conclusion

Using the implicit leadership–imitation framework as the analytical lens, this study suggests an underlying structural pattern of informal influence within groups of university students. In contrast to traditional perspectives that position leadership and conformity as opposing forces, the findings indicate that social learning among university students is not merely a process of compliance to authority. Instead, it can be understood as a dynamic pattern associated with cognitive processing, behavioral imitation, and emotional resonance. This finding enhances the current understanding of peer influence and learning motivation and provides a useful perspective for interpreting group dynamics in higher education contexts.

### 7.1 Theoretical contributions: embedding implicit leadership into learning sciences

At the theoretical level, this study integrates ILT with SLT to propose a conceptual framework for decentralized leadership and a multilevel imitation system. The results suggest that, within informal groups, leadership-related processes are not dependent on formal positional structures; instead, they are associated with the perceptions of group members regarding leadership prototypes. This pattern aligns with recent scholarship on shared leadership and informal influence (Carson et al., 2007; DeRue et al., 2011).

These studies collectively suggest that leadership processes do not need to originate from authoritative positions but can be associated with cognitive alignment, role modeling, and social referential processes (Lord et al., 1984; Shondrick et al., 2010). The present study further supports this perspective in an educational context: implicit leaders among student groups do not possess formal power, but they may function as salient reference points for others by serving as behavioral exemplars, cognitive strategies, and emotional attunement. In doing so, this study extends the concept of implicit leadership from organizational psychology to the domain of learning sciences, thus enriching its applicability in decentralized social learning contexts.

### 7.2 Methodological innovation: a three-layer validation framework of measurement, structure, and group differences

Methodologically, this study constructs a continuous validation system that integrates reliability and validity testing; factor and principal component analyses; and SEM to reveal latent dimensions and structural relationships. Through the coordinated application of EFA (as the primary factor extraction method using principal axis factoring), PCA (as an auxiliary technique for examining variance distribution), and SEM, it establishes a comprehensive logical chain from measurement instruments to structural models—effectively controlling for risks of measurement redundancy and structural inflation (Kline, 2016).

This tripartite paradigm—linking measurement, structure, and group differences—provides a replicable methodological pathway for future research that intends to integrate implicit influence with social network indicators, behavioral logs, and multimodal classroom data (D'Mello and Graesser, 2015).

### 7.3 Educational implications: activating decentralized influence within learning groups

From the educational practice perspective, this study reveals a new logic of *learning influence* in higher education. First, instructors or peer mentors may explicitly demonstrate effective learning strategies (e.g., time management and task decomposition) in classroom settings, thus making these strategies more observable to students can perceive and imitate. Prior research indicates that deliberate demonstrations of learning strategies and spaced practice can significantly enhance students’ internalization and application of these strategies (Carpenter et al., 2012).

Second, by incorporating rotating leadership roles and justification-sharing mechanisms into group tasks, educators can strengthen shared interpretive processes and facilitate the transformation of cognitive alignment into consensus in team decision-making. This approach is consistent with the research on team effectiveness that emphasizes the role of process mechanisms and environmental structures (Mathieu et al., 2019).

Finally, with respect to emotion, training in emotional expression and structured peer feedback may help improve teams’ affective climates, enabling emotional synchrony to function as a facilitator of coordination rather than a source of pressure. Although direct experimental evidence for this pathway remains limited, the existing research on academic emotion measurement provides a methodological foundation for future classroom interventions (Govaerts and Grégoire, 2008). Based on these insights, efforts to promote student autonomy and collaboration should, therefore, focus on the activation of internal learning signals and social imitation mechanisms within groups, instead of solely relying on traditional forms of authoritative control.

### 7.4 Cultural and social implications: relational learning mechanisms in east Asian contexts

From cultural and social perspectives, this study provides empirical support for understanding collectivist leadership in East Asian contexts. The imitative behaviors of Chinese university students are not merely passive conformity; instead, they can be interpreted as a form of social identification associated with cognitive alignment and emotional resonance. This cognition-oriented and emotion-related pattern of social learning reflects characteristics of the relational self and affective attunement commonly associated with East Asian cultures. It also suggests that leadership and learning are closely intertwined within collectivist contexts.

## Data Availability

The datasets presented in this study can be found in online repositories. The names of the repository/repositories and accession number(s) can be found at: https://github.com/a359738533/Dataset_IL.
